# Ligand-Field and Spin–Orbit Coupling Competition in a Pb−π Model

**DOI:** 10.1021/acs.jpca.6c02546

**Published:** 2026-08-11

**Authors:** Fahri Alkan, Sefik Suzer, Paul S. Bagus

**Affiliations:** † 52948 Bilkent University, Chemistry Department, 06800 Ankara, Turkey; ‡ Center for Advanced Scientific Computing and Modeling (CASCaM), Department of Chemistry, 3404 University of North Texas, 1155 Union Circle #305070, Denton, Texas 76203-5017, United States

## Abstract

There has been a strong recent interest in the large spin–orbit coupling (SOC), a relativistic effect, in Pb moieties adsorbed on graphene and related 2D materials. Since the SOC mainly originates from the strong nuclear potential of heavy atoms, a conceptually simple approach is adopted to relate the underlying physical/chemical origin of the large spin–orbit splitting to the lead atom’s outermost valence 6p atomic level. To examine this possible origin at the most fundamental level, we investigated a minimal heavy-adatom−π model system consisting of a single Pb atom positioned at various distances above the center of a benzene (C_6_H_6_) molecule. In order to analyze the evolution of the electronic structure of this model system, fully relativistic four-component Dirac–Hartree–Fock (4c-DHF) calculations, which took account of the angular momentum coupling of the open shell, Pb 6p, electrons, as well as quasi-relativistic approaches are used. Our results show that the ligand field effects exerted by benzene on the excited states of Pb are comparable in magnitude to the intrinsic 6p SOC of the Pb atom when it is at short distances above the C_6_H_6_. Indeed, in this regime, the SOC and ligand-field interactions cannot be cleanly separated. In addition, single-determinant and quasi-relativistic SOC treatments are shown to overestimate the Pb–benzene interaction and the associated SOC transfer to the π system, whereas the inclusion of multiconfigurational effects significantly reduces SOC within the open-shell space. A key result of our findings is that the SOC in heavy-adatom−π systems is not a simple projection of atomic SOC, but rather a consequence of hybridization, which depends critically on the correlated description of the heavy-atom valence shell.

## Graphical abstract

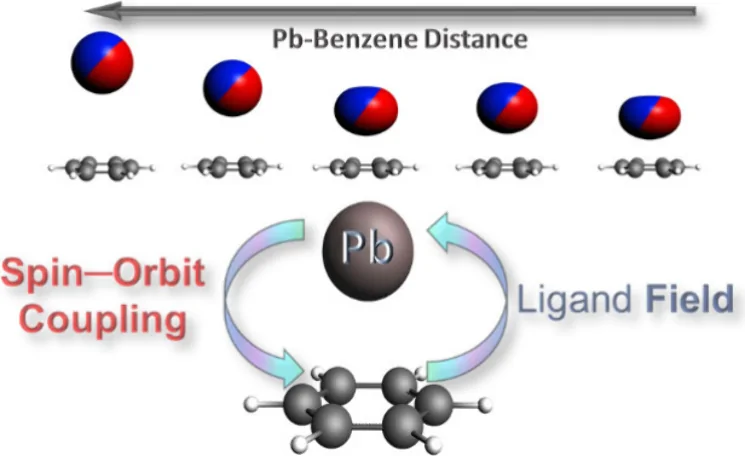

## Introduction

Spin–orbit coupling (SOC) is a relativistic phenomenon that grows sharply with the atomic number, Z.^1–7^ As a result of this Z dependence, SOC splittings range from meV scale for 2p atoms, up to several eV in the valence shells of heavier 6p atoms.^8,9^ For example, the multiplets for the 2p^2^ configuration of the C atom are best described as Russell–Saunders ^3^P, ^1^D, and ^1^S with small spin–orbit splittings of the ^3^P multiplet. However, for Pb, the multiplets are best described as configurations with occupations 6p_1/2_^2^, 6p_1/2_6p_3/2_, and 6p_3/2_^2^, with further splitting into different total angular-momentum couplings.^1^

These observations might suggest that SOC only needs to be considered when dealing with the electronic structure or spectroscopy of heavy elements.^5,6,10–13^ In reality, SOC is also relevant for light atoms or light molecules when they are interacting with heavy atoms. Perhaps the most apparent of such cases is for the NMR chemical shifts of the heavy atom effect on light atom shielding, described as HALA effects.^14–17^ For example, it has been shown by Seamanet al.^18^ that a more than +300 ppm shift is observed for the ^13^C NMR of uranium-bonded methylene in a U^VI^-hexaalkyl complex. Another important example is the heavy atom effect in photophysical and photochemical processes.^19,20^ In recent years, heavy-atom-induced SOC has been strategically exploited to enhance intersystem crossing (ISC) and reverse ISC,^21–23^ thereby improving the performance of applications such as organic light-emitting diodes (OLEDs), photodynamic therapy (PDT), triplet harvesting in solar cells, and molecular spintronic devices.^20,24^

In recent years, there has been growing interest in SOC-enhanced properties of surfaces and interfaces and two-dimensional (2D) materials.^25^ In pristine graphene, SOC is theoretically and experimentally demonstrated to be quite small, as expected.^26,27^ However, SOC can be significantly enhanced through the introduction of heavy adatoms, either via proximity effects or through direct hybridization.^28–33^ For example, in a seminal study by Marchenko et al.,^34^ it was shown that strong graphene–gold hybridization can induce a substantial SOC (around 100 meV) in the graphene. Similarly, large SOC enhancements have been observed for Pb atom adsorbates.^35,36^ Theoretical investigations have also been employed to elucidate the interaction mechanisms between adatoms and the graphene lattice, offering insight into how the SOC is induced as a function of parameters such as graphene–adatom distance, adsorption position, and adatom concentration.^35,37^ Nonetheless, these computational studies generally rely on perturbative treatments of the SOC Hamiltonian,^38–44^ and a comprehensive interaction picture based on a fully relativistic approach remains absent from the literature.

In the 1970s, it was established using both purely theoretical and experimental work that the J = 0 ground state of the Pb atom favors a significant population of the 6p_1/2_ sublevel due to the large SOC.^1–4^ Since a combination of electrostatic, magnetic, and van der Waals forces is involved when molecules and/or solids are formed, one can ask the question: “What atomic relativistic effects exactly have been preserved and to what extent, in going from the isolated gas phase Pb atoms to molecules and/or solids containing them?” In order to understand the interaction between a Pb atom and a π-system, we performed quantum chemical calculations on a Pb–C_6_H_6_ (Pb–benzene) model using various levels of theory incorporating SOC. Our results reveal that the apparent transfer of SOC from heavy to light atoms arises from the formation of covalent bonding and antibonding orbitals between Pb and the benzene π-system. Indeed, when this interaction is sufficiently strong, separating the spin–orbit splitting from the ligand-field interaction is impossible. Moreover, the magnitude of Pb–benzene interaction is highly sensitive to the treatment of SOC. These findings offer new insights into adatom−π-system interactions from a relativistic quantum chemistry perspective.

## Computational Methods

4c-DHF (Dirac Hartree–Fock) calculations were carried out using the DIRAC program package,^45^ ZORA/CIS (Zeroth Order Regular Approximation with Configuration Interaction Singles) calculations were performed with ADF 2023.^46,47^ In the case of 4c-DHF calculations, an uncontracted Dyall all-electron triple-ζ (pVTZ) Gaussian basis set^48,49^ with a minor modification to the most diffuse polarization function was employed for Pb, whereas the Gaussian basis sets of van Duijneveldt^50^ augmented with the polarization functions described in ref 51 were used for C and H. The complete set of basis-set exponents used in the present work is provided in the Supporting Information to ensure full reproducibility. In the case of ZORA/CIS calculations, an all-electron triple-ζ-double-polarization (TZ2P) basis set was used for all atoms as implemented in ADF.^52^

For 4c-DHF wave functions, a COSCI (complete open-shell configuration interaction), formulation was used in order to have wave functions that belong to appropriate multiplets with suitable degeneracies. In effect, this is needed to properly treat the interaction of the two open-shell 6p electrons. The active orbital space was the six dominantly Pb 6p spin orbitals, and the COSCI determinants were formed by distributing 2 electrons in all possible ways over these active orbitals. For this case, the scalar and spin–orbit effects were taken into account self-consistently with the Dirac–Coulomb Hamiltonian. To study how properties change between fully relativistic Dirac calculations and parallel nonrelativistic calculations, we have modified the speed of light, denoted as c. The measured speed of light,^53^ in atomic units, c = 137.035998 is used in our fully relativistic Dirac calculations. To obtain nonrelativistic results with exactly parallel basis set parameters, we carried out Dirac calculations with c = 25,000 atomic units. We have further verified that increasing c does not change our Dirac results to the decimal place displayed for all properties. In the case of ZORA/CIS, the ground state wave function was calculated with the ZORA-Hartree–Fock method with the spin–orbit term included, and excited states were calculated at the CIS level. The Pb–benzene geometries were constructed by placing the Pb atom along the 6-fold symmetry axis above the center of the benzene ring at fixed Pb–benzene separations,d, with values of d between 5.0 and 2.5 Å, as illustrated in Figure 1. These distances were chosen to span weak-perturbation to strong-interaction regimes; see Tables of orbital and wave function properties in the Supporting Information (SI) section. Throughout this work, the conventional spectroscopic labels (^3^P_0_, ^3^P_1_, ^3^P_2_, etc.) are used as only correlation labels for the low-lying atomic states of Pb and do not imply pure Russell–Saunders coupling.

**Figure 1:**
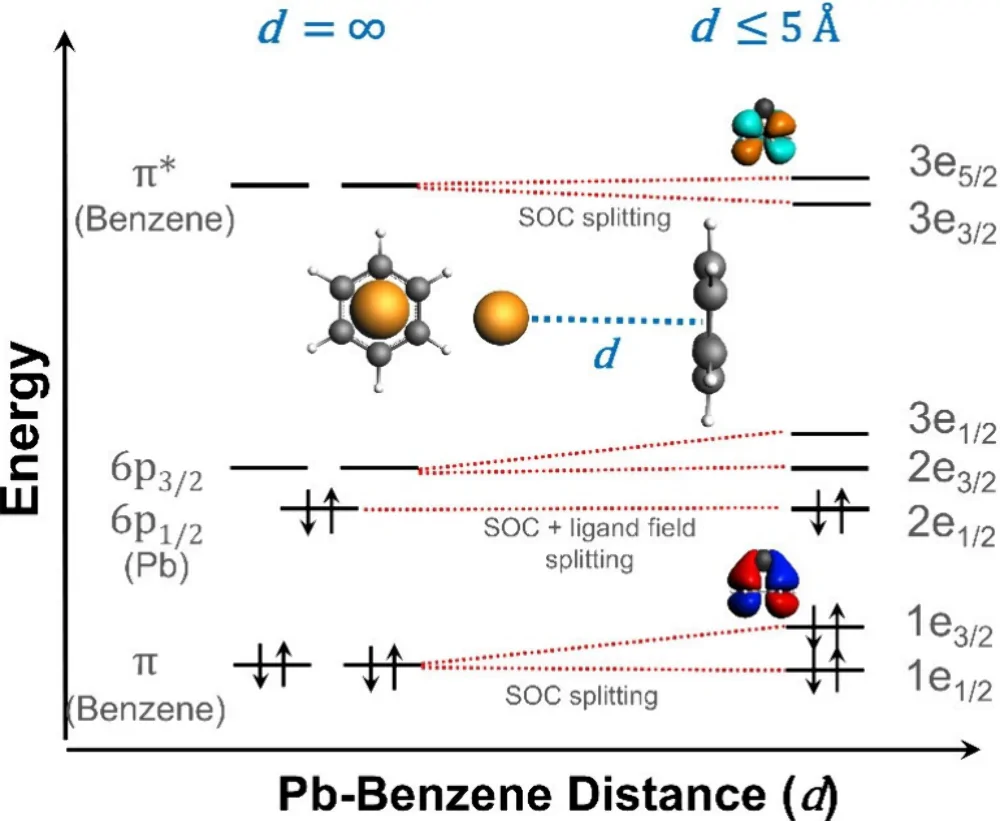
Schematic interaction picture for the Pb-Benzene orbitals from an orbital energy level perspective. Herein, *d*=∞ corresponds to isolated Pb and benzene. Upon interaction at finite d, the Pb 6p levels are split by both SOC and ligand field, whereas the splitting of benzene π and π* is solely due to the SOC induced by the mixing of Pb 6p and π or π* levels. The symmetry labels of the spinors for the interacting case (1*e*_1/2_, 1e_3/2_, ...) refer only to the subset of levels that originate from Pb 6p, and benzene π and π* levels.

## Results and Discussion

The interaction between the Pb atom and the benzene molecule is determined mainly by three factors: the orbital mixing between Pb 6p_x,y_, and benzene π or π* levels, the repulsion interaction between 6p_*z*_ and benzene, and the SOC interaction. In Figure 1, we illustrate this description of the interaction using one-electron energy levels and group theory arguments. In the schematic of Figure 1, we show the orbital energies and symmetries of the relevant Pb and C_6_H_6_ orbitals for large separation, denoted as d = ∞, compared to smaller separation, denoted as *d* ≤ 5Å. The π orbitals (E_1_) split into *e*_1/2_ and e_3/2_ in the C_6v_* double group, while the π* (E_2_) orbitals split into e_3/2_ and e_5/2_, the spin–orbit split Pb 6p_1/2_ goes to *e*_1/2_, and the 6p_3/2_ goes to e_3/2_ and e_1.2_ in C_6v_*. In the j-j coupling limit with strong SOC, an isolated Pb atom (d = ∞) has a ground J = 0 state with the closed shell configuration of 6p^2^_1/2_ 6p^0^_3/2_, while benzene’s electronic structure has doubly degenerate π (HOMO) and π* (LUMO) levels. Upon interaction (*d* ≤ 5 Å), these levels split according to C_6v_ double-group symmetry, where 6p levels split into 2*e*_1/2_, 2e_3/2_, and 3*e*_1/2_. Meanwhile, π and π* levels split into 1*e*_1/2_-1e_3/2_ and 3e_3/2_-1e_5/2_. We note that without SOC, benzene-originated π and π* levels are 4-fold degenerate under C_6v_ symmetry, and the broken degeneracy of these levels originates solely from SOC, which is mainly enhanced by mixing with Pb 6p levels.

While the interaction picture shown in Figure 1 provides a qualitative understanding of the energetics from an orbital perspective and symmetry arguments, it does not take the multiconfigurational character of the wave functions into account, where the wave functions are mixtures of configurations with different occupations of the 6p orbitals. Meanwhile, it is shown both experimentally and theoretically that, while being closer to the j-j limit shown in Figure 1, the ground state of Pb exhibits some multiconfigurational character, where the occupation of 6p_1/2_ is 1.86 (not 2.0 as in the simple model) and the 6p_3/2_ occupation is 0.14 (not 0).^3,54^ This is a clear indication that at least a limited multiconfigurational approach is necessary to describe this 2-electron open-shell system. Furthermore, the multiconfiguration character is also observed for the excited states of the Pb–C_6_H_6_ complex (*vide infra*).

To analyze the interplay between ligand-field and SOC effects, we performed 4c-DHF calculations with a complete active space configuration interaction (4c-DHF-COSCI) treatment of the two 6p electrons of Pb over the spin–orbit split C_6v_* e orbitals, see Figure 1. Wave functions were obtained for the ground and excited states of the Pb-benzene complex at several Pb-benzene distances (d = 5.0, 4.0, 3.0, 2.75, and 2.5 Å). The results are illustrated in Figures 2a and 2b for the fully relativistic case with the physical speed of light, and the spin-free nonrelativistic case is modeled with a very large value of c = 25,000 au The numerical values for the plots in Figure 2 are given in the SI, Tables S1 and S2. For isolated Pb, the ground state corresponds to the J = 0 case (^3^P_0_), and the energy differences between the ground state and the states with J = 1 (^3^P_1_) and J = 2 (^3^P_2_) are calculated to be 0.86 and 1.33 eV, compared to experimental values of 0.97 and 1.32 eV.^3^

**Figure 2:**
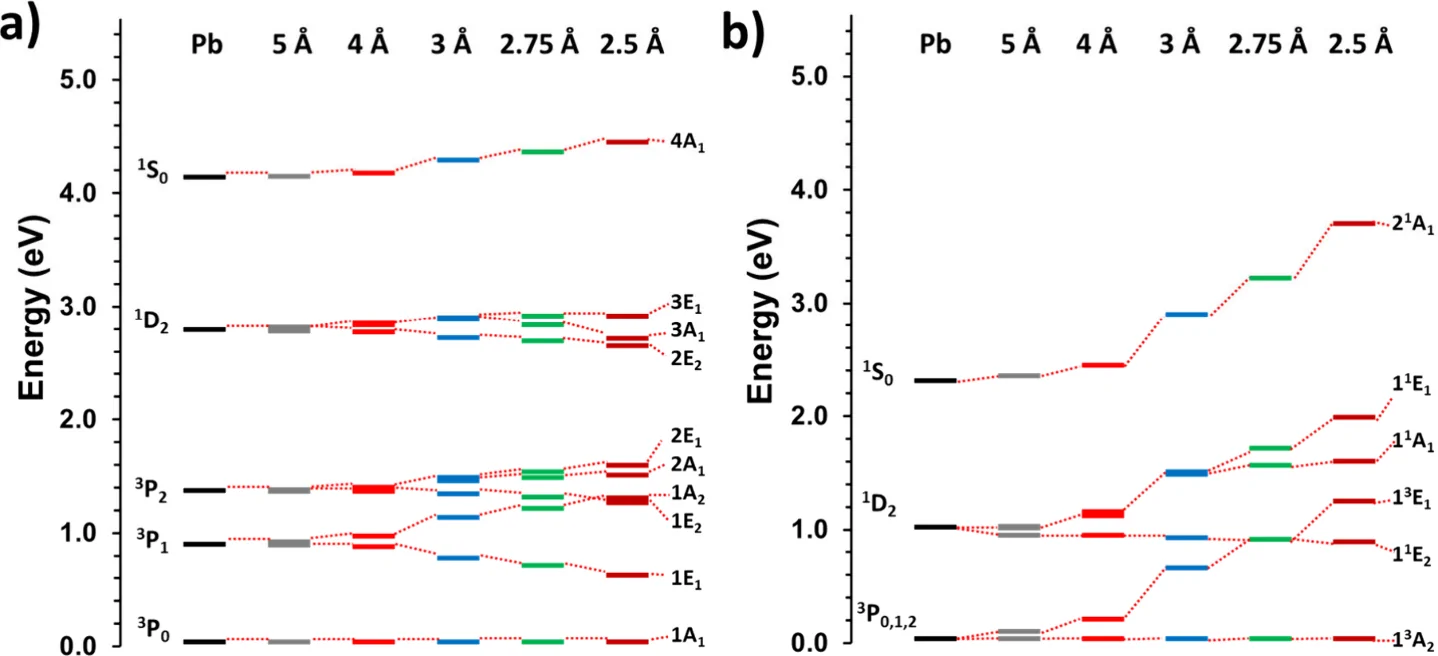
Calculated excited state energy splittings for isolated Pb and Pb-benzene systems (*d* = 5.0–2.5 Å) with open-shell CI formalism of 4-component Dirac Hartree–Fock Hamiltonian. In (a), splittings are illustrated for the physical value of c, whereas (b) shows the nonrelativistic splittings obtained with c = 25,000 au.

In the presence of the benzene ring, the electronic structure exhibits a competition between the ligand field and the SOC. With 4c-DHF-COSCI, the states are most appropriately discussed in terms of C_6v_ double-group symmetry. In this work, we use Mulliken notation for the double-group irreducible representations, while an alternative Bethe notation is also used in the literature.^55^ In this case, the ground state transforms to 1A_1_ state, triply degenerate ^3^P_1_ splits into a doubly degenerate 1E_1_ and a singly degenerate 1A_2_ state, and ^3^P_2_ splits into a doubly degenerate 1E_2_, a doubly degenerate 2E_1_, and a singly degenerate 2A_1_ state. A similar splitting is seen for the higher-lying ^1^D_2_ state (3A_1_-2E_2_-3E_1_), whereas the ^1^S_0_ (4.10 eV) transforms to 4A_1_. At d = 5.0 or 4.0 Å, the benzene ligand field induces only minor splittings (0.01–0.10 eV) to the Pb-centered excited-state manifolds, indicating a weak perturbation regime. As the Pb–benzene distance decreases, these splittings increase significantly due to enhanced orbital mixing and covalent and/or repulsive interactions. At a distance of 2.5 Å, the calculated splittings become 0.68, 0.33, and 0.26 eV for the energy levels originating from ^3^P_1_, ^3^P_2_, and ^1^D_2_ levels, respectively. Overall, the ligand-field induced energy splittings become comparable to the intrinsic SOC splittings of the Pb 6p shell for *d* ≤ 3 Å.

In addition to 4c-DHF calculations, we have performed complementary ZORA/CIS calculations to further examine the interplay between SOC and ligand field. Since quasirelativistic approaches such as ZORA are routinely employed for heavy-adatom and extended π-conjugated systems, this comparison provides insight into their reliability for Pb-containing systems where relativistic and multiconfigurational effects are significant. Due to the single-excitation character of ZORA/CIS, only the ^3^P manifold with J = 0,1, and 2 states are predicted (Table S3). It is also noted that the reference ground-state wave function has single-determinant character with a 6p_1/2_^2^ 6p_3/2_^0^ electron configuration. In this case, the intrinsic SOC splitting between ^3^P_0_ and ^3^P_1_ states (0.52 eV) is considerably lower compared to the 4c-DHF value (0.86 eV), indicating the approximate nature of the two-component ZORA treatment of spin–orbit coupling. These values of the splitting can be seen from the d = 5.0 ^3^P_0_^–3^P_1_ splittings as shown in Figure 3a, since the splittings at this large distance are essentially those of isolated Pb. This underestimation is persistent for different Pb-benzene distances, as shown in Figure 3a. However, the ligand-field-induced splittings within the ^3^P_1_ and ^3^P_2_ manifolds are reproduced with very good agreement, as shown in Figures 3b and 3c. Thus, although the SOC magnitude is underestimated, the growth of ligand-field splittings with decreasing distance is consistently captured with ZORA/CIS as well, consistent with the emergence of SOC–ligand-field competition.

**Figure 3:**
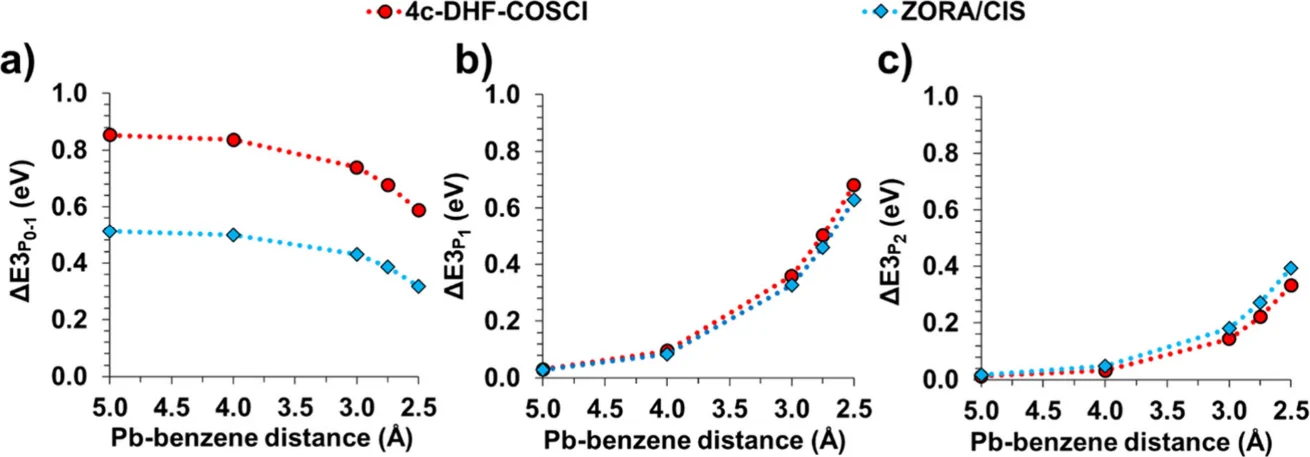
a) SOC splitting between^3^P_0_ and ^3^P_1_ states, b) ligand-field induced splitting of the ^3^P_1_ manifold, and c) ligand-field induced splitting of the ^3^P_2_ manifold calculated with 4c-DHF-COSCI and ZORA/CIS formalisms for varying Pb-benzene distances.

To further understand the orbital origin of the trends observed in Figures 2 and 3, we analyze the evolution of the Pb 6p spinor character and angular-momentum composition in Table 1 using 4c-DHF-COSCI (fully relativistic). The same analysis in the absence of SOC (c = 25,000 au) is provided in Table 2. In these tables, we give the < l_*z*_>, < s_*z*_>, and < j_*z*_> for three of the 6 open shell spinors. Recall that there is a double degeneracy for the Kramers pairs in the DHF formulation, and we choose the spinors where the < j_*z*_> is positive; clearly for the other member of the Kramers pair, the signs of the angular momentum expectation values are reversed while the magnitudes are identical. For these spinors, the Mulliken populations in terms of p_*x*_, p_*y*_, and p_*z*_ are also given; these populations are identical for the Kramers pair, which is not shown. For the isolated Pb, the calculated ⟨l_*z*_⟩, ⟨s_*z*_⟩ and ⟨j_*z*_⟩ values are consistent with the well-defined 6p_1/2_ and 6p_3/2_ spinors with a splitting of 1.25 eV, and the orbitals are denoted as 6p_1/2_ and 6p_3/2_, where two degenerate components exhibit j_*z*_ = 3/2 and 1/2, respectively. We also note that the lowest-energy 6p_1/2_ spinor shows a complete isotropi≈character in terms of the corresponding Cartesian populations of 6p_*x*_, 6p_*y*_, and 6p_*z*_. For the C_6v_* double group, J and j_*z*_ are no longer good quantum numbers, but the nomenclature 6p_1/2_ and 6p_3/2_ continue to be used for the two longer distances of Pb above C_6_H_6_, d = 5.0 and d = 4.0, we continue to use the j_*z*_ notation for the orbitals, although there is a modest splitting of the two “6p_3/2_” orbitals. For smaller distances, d, the splitting and mixing of these becomes so large that we drop the designations of 1/2 and 3/2 and simply describe the orbitals as 6p_1_, 6p_2_, and 6p_3_.

**Table 1:** Orbital Energies, Angular Momentum Expectation Values, and Populations for Pb 6p_1/2_ and 6p_3/2_ Energy Levels for Isolated Pb and Pb-Benzene Complexes Calculated with 4c-DHF-COSCI (Fully Relativistic)

		Angular momentum			Populations		
Orbital	Energy (eV)	⟨l_*z*_⟩	⟨s_*z*_⟩	⟨j_*z*_⟩	6p_*x*_	6p_*y*_	6p_*z*_
Isolated Pb							
6p_1/2_	–2.64	0.667	–0.167	0.500	0.333	0.333	0.333
6p_3/2_	–1.39	1.000	0.500	1.500	0.024	0.380	0.596
6p_3/2_	–1.39	0.333	0.167	0.500	0.642	0.287	0.071
Pb-benzene d = 5 Å							
6p_1/2_	–2.47	0.687	–0.187	0.500	0.344	0.344	0.313
6p_3/2_	–1.23	1.000	0.500	1.500	0.500	0.500	0.000
6p_3/2_	–1.18	0.313	0.187	0.500	0.157	0.157	0.689
Pb-benzene d = 4 Å							
6p_1/2_	–2.29	0.717	–0.217	0.500	0.359	0.359	0.284
6p_3/2_	–1.08	0.999	0.500	1.499	0.501	0.501	0.000
6p_3/2_	–0.95	0.284	0.216	0.500	0.142	0.142	0.724
Pb-benzene d = 3 Å							
6p_1_	–1.70	0.799	–0.303	0.495	0.399	0.399	0.196
6p_2_	–0.59	0.994	0.500	1.494	0.503	0.503	0.000
6p_3_	–0.19	0.197	0.301	0.499	0.100	0.100	0.810
Pb-benzene d = 2.75 Å							
6p_1_	–1.42	0.828	–0.336	0.492	0.414	0.414	0.161
6p_2_	–0.36	0.991	0.500	1.491	0.505	0.505	0.000
6p_3_	0.17	0.164	0.334	0.499	0.085	0.085	0.827
Pb-benzene d = 2.5 Å							
6p_1_	–1.07	0.858	–0.371	0.487	0.430	0.430	0.123
6p_2_	–0.08	0.987	0.500	1.487	0.509	0.509	0.000
6p_3_	0.58	0.130	0.369	0.498	0.069	0.069	0.826

**Table 2:** Orbital Energies, Angular Momentum Expectation Values, and Populations for Pb 6p_1/2_ and 6p_3/2_ Energy Levels for Isolated Pb and Pb-Benzene Complexes Calculated with 4c-DHF-COSCI (c = 25,000 a.u.)

		Angular momentum			Populations		
Orbital	Energy (eV)	⟨l_*z*_⟩	⟨s_*z*_⟩	⟨j_*z*_⟩	6p_*x*_	6p_*y*_	6p_*z*_
Isolated Pb							
6p_1_	–1.97	1.000	–0.500	0.500	0.500	0.500	0.000
6p_2_	–1.97	–1.000	–0.500	–1.500	0.500	0.500	0.000
6p_3_	–1.97	0.000	0.500	0.500	0.000	0.000	1.000
Pb-benzene d = 5 Å							
6p_1_	–1.82	0.915	–0.500	0.415	0.299	0.702	0.000
6p_2_	–1.82	–0.915	–0.500	–1.415	0.702	0.299	0.000
6p_3_	–1.75	0.000	0.500	0.500	0.000	0.000	1.002
Pb-benzene d = 4 Å							
6p_1_	–1.66	0.894	–0.500	0.394	0.277	0.724	0.000
6p_2_	–1.66	–0.894	–0.500	–1.394	0.724	0.277	0.000
6p_3_	–1.47	0.000	0.500	0.500	0.000	0.000	1.009
Pb-benzene d = 3 Å							
6p_1_	–1.10	0.899	–0.500	0.399	0.287	0.715	0.000
6p_2_	–1.10	–0.899	–0.500	–1.399	0.715	0.287	0.000
6p_3_	–0.49	0.000	0.500	0.500	0.000	0.000	1.021
Pb-benzene d = 2.75 Å							
6p_1_	–0.84	0.952	–0.500	0.452	0.364	0.642	0.000
6p_2_	–0.84	–0.952	–0.500	–1.452	0.642	0.364	0.000
6p_3_	–0.01	0.000	0.500	0.500	0.000	0.000	1.014
Pb-benzene d = 2.5 Å							
6p_1_	–0.53	–0.885	0.965	–0.500	0.610	0.404	0.000
6p_2_	–0.53	–0.885	–0.965	–0.500	0.404	0.610	0.000
6p_3_	0.58	–0.951	0.000	0.500	0.000	0.000	1.000

As we noted above, upon interaction with benzene, this isotropy of the 6p_1/2_ is lifted somewhat for the d = 5 and 4 Å separation distances. This effect is illustrated directly in Figure 4, which shows the evolution of the Pb 6p_*x*_, 6p_*y*_, and 6p_*z*_ populations of the occupied level as a function of Pb–benzene distance. The benzene ring defines a unique molecular axis (the *z*-axis), and the repulsive interaction between the Pb 6p_*z*_ component and the closed-shell π system induces a directional polarization for the 6p orbitals. In the fully relativistic regime (Table 1), the combination of ligand field and SOC distributes the 6p_*z*_ character among the spinors, where the lowest-energy (most negative) spinor shows a continuous reduction (from 0.313 at d = 5.0 Å to 0.123 at d = 2.5 Å) in 6p_*z*_ population and the highest-energy spinor shows an increasing 6p_*z*_ population (0.689→0.826). This trend is consistent with the behavior observed in Figure 4, where the 6p_*z*_ contribution decreases systematically with decreasing distance, while the in-plane components increase. In addition, the expectation value of j_*z*_ deviates from atomic limits (i.e., decreases), especially for the cases where d ≤ 3Å, indicating that the Pb-centered spinors are no longer well described by pure 6p_1/2_ and 6p_3/2_ character as the interaction enters the mixing regime. These findings also call for caution when interpreting the interaction solely in terms of Pb 6p_*z*_ orbital interaction with the carbon orbitals, since SOC reduces orbital directionality, hence weakening the conventional picture of directional bonding.^4,5^ We also note that the ligand-field induced redistribution of orbital character strongly affects the spin–orbit coupling and chemical interaction, as changes in orbital composition alter both the angular-momentum character and the extent of Pb−π hybridization.

**Figure 4:**
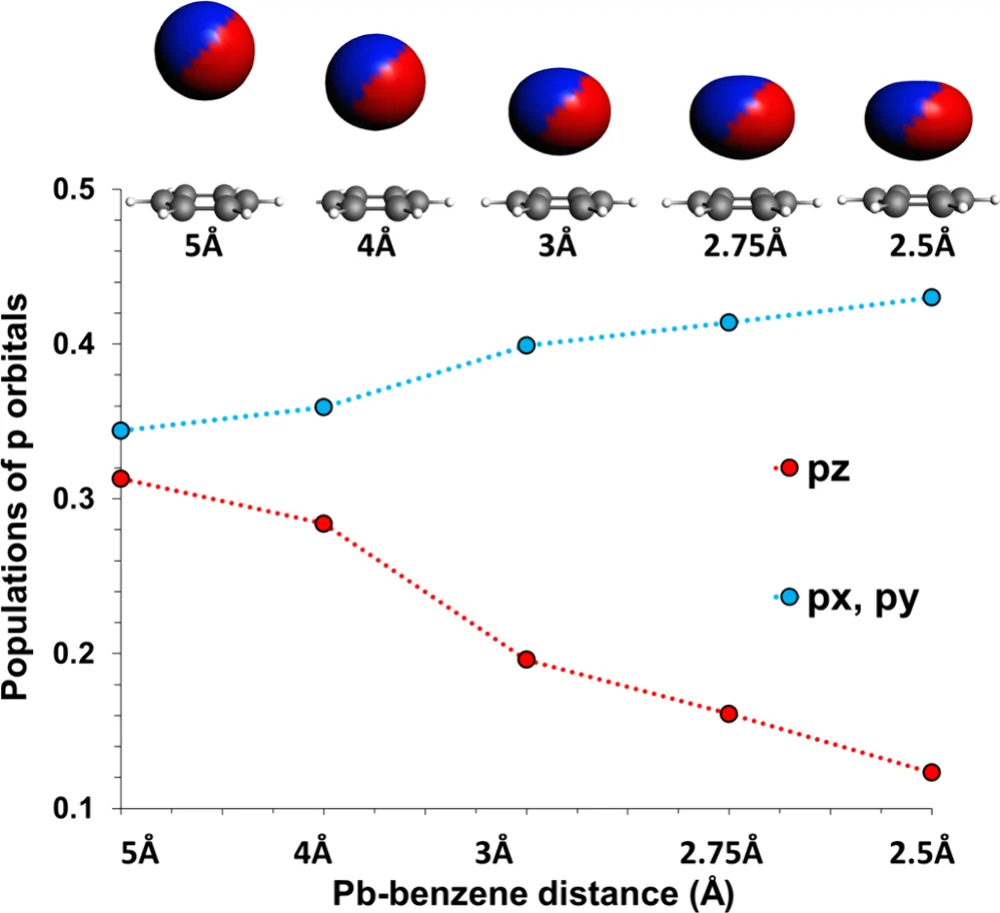
Illustration of the Pb-centered occupied level with Pb 6p_*x*_, 6p_*y*_, and 6p_*z*_ populations vs Pb–benzene distance, showing the loss of isotropy. The orbital populations shown here were obtained from the 4c-DHF-COSCI wave functions.

In the nonrelativistic, spin-free limit (Table 2), the ligand-field splitting of the 6p orbitals follows directly from C_6v_ point-group symmetry. In this case, the 6p_*z*_ orbital transforms as the singly degenerate A_1_ representation, whereas the 6p_x, y_ transform as the doubly degenerate E_1_ representation. In that regard, the highest-energy orbital becomes a pure 6p_*z*_ orbital (A_1_) due to the repulsive interaction, whereas the low-energy pair exhibits a mixed 6p_*x*_ - 6p_*y*_ (E_1_) character. We also note that the magnitude of the ligand-field–induced splitting of orbitals in the spin-free case is comparable to the SOC-induced reorganization observed in the fully relativistic treatment. This demonstrates that, in the Pb–benzene interaction picture, ligand-field effects and spin–orbit coupling operate on similar energy scales and must be treated simultaneously to obtain a balanced description of the evolving 6p spinor character.

In addition to states derived from Pb atomic levels, we now examine how the benzene π molecular orbitals (1e_1/2_-1e_3/2_ in Figure 1) respond to the presence of strong spin–orbit coupling induced by the Pb atom. For isolated benzene, the π orbitals are doubly degenerate and SOC-induced splitting is negligible, consistent with the light-atom character of carbon. Accordingly, in the absence of Pb, the splitting of the π levels is calculated to be 0.002 eV. Upon interaction with Pb, this splitting increases systematically with decreasing Pb-benzene distance, as shown in Figure 5, due to the mixing of Pb 6p and benzene π levels. Here, the j–j coupling limit corresponds to a closed-shell 6p_1_/_2_^2^ configuration of the Pb atom, whereas the configuration-mixed case refers to the multiconfigurational redistribution of the 6p electrons across the orbitals. We also note that the benzene π orbitals are not included in the active space and their energies are affected mainly through Pb−π hybridization.

**Figure 5:**
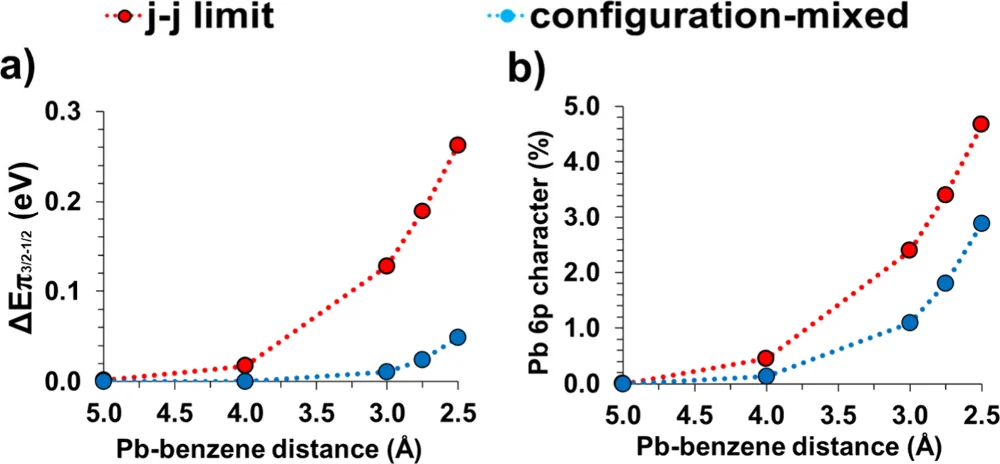
(a) The spin–orbit splitting of benzene-derived π levels (1e_1/2_-1e_3/2_) as a function of Pb–benzene distance, resulting from hybridization with Pb 6p orbitals. The two curves correspond to the closed-shell 4c-DHF reference (j–j limit) and the multiconfigurational 4c-DHF-COSCI treatment, respectively. (b) Pb 6p contribution to the corresponding 1e_1/2_-1e_3/2_ molecular orbitals, expressed as Mulliken populations.

Within the j–j coupling limit (as described by the 4c-DHF treatment), the splitting grows from essentially zero at 5 Å to 0.26 eV at 2.5 Å, in direct correspondence with the increasing Pb contribution to the π orbitals (from 0% to 4.67%). The agreement between SOC splitting and Pb contribution indicates that the effective SOC transferred to the π system is governed by the extent of Pb-π hybridization. We note that nearly identical behavior for the SOC splitting of π orbitals can be obtained with the quasi-relativistic ZORA Hamiltonian using CIS formalism, as shown in Figure S1 and Table S4. Notably, this splitting is also comparable to the SOC-induced gap at the Dirac point of graphene upon Pb adsorption at a similar distance, as reported by Brey and Calleja et al.^35^

An important difference emerges when multiconfigurational effects are included. In the j-j limit (4c-DHF reference or ZORA calculations), the Pb 6p shell adopts a closed-shell occupation, corresponding to 6p_1/2_^2^ configuration. In contrast, the multiconfigurational wave function redistributes the two 6p electrons across the spinors. This configurational mixing partially reduces the effective SOC transferred to the benzene π orbitals. Consequently, the π-level splitting predicted is substantially smaller (0.05 eV at 2.5 Å, with 2.88% Pb character) than the j-j limit. These results demonstrate that the SOC observed in the π system is not an intrinsic property of benzene, but arises entirely from Pb−π hybridization, and that its magnitude depends significantly on the correlated description of the Pb 6p orbitals.

## Conclusion

In summary, we have examined the ground and excited states of the Pb–benzene complex using fully relativistic 4c-DHF-COSCI and quasi-relativistic ZORA/CIS treatments of SOC. Our results demonstrate that the ligand-field effects exerted by benzene on the Pb 6p manifold become comparable in magnitude to the intrinsic SOC splittings at short Pb–benzene separations. In this regime, SOC and covalent interactions within the Pb 6p manifold are intrinsically intertwined and cannot be treated as separable perturbations. Furthermore, approaches that neglect the multiconfigurational character of the Pb 6p shell tend to overestimate the effective Pb–benzene interaction and the apparent SOC transfer to the π system. The correlated 4c-DHF-COSCI description reveals that configurational mixing within the Pb valence shell significantly moderates the induced SOC in the π system. These findings show that SOC in heavy-adatom−π systems is not simply inherited from the atomic splitting of the heavy element, but emerges from hybridization whose magnitude depends critically on a balanced relativistic and multiconfigurational treatment.

## Supplementary Material



## Notes

Detailed analyses of spinor character, SOC-induced splittings, and comparisons between 4c-DHF-COSCI and ZORA/CIS results (PDF)
