# Supplementary material for: Ligand-Field and Spin–Orbit Coupling Competition in a Pb−π Model
Source: J Phys Chem A. 2026 Aug 11;130(34):6699–706. doi: 10.1021/acs.jpca.6c02546 (PMC13528297; doi:10.1021/acs.jpca.6c02546)
Supplement: Supplementary file 1 [file jp6c02546_si_001.pdf]

Supporting Information for

Ligand-Field and Spin–Orbit Coupling Competition in a Pb–π Model

Fahri Alkan[1], Sefik Suzer[1] and Paul S. Bagus[2]

[1]Bilkent University, Chemistry Department, 06800 Ankara, Turkey

[2]Center for Advanced Scientific Computing and Modeling (CASCaM), Department of Chemistry University of North Texas, 1155 Union Circle #305070, Denton, Texas 76203-5017, USA

Table S1. Excited-state energies and term symbols for isolated Pb and Pb-benzene complex at various distances calculated with the 4c-DHF method (fully relativistic)

**Isolated Pb**		**Pb-benzene d=5Å**		**Pb-benzene d=4Å**		**Pb-benzene d=3Å**		**Pb-benzene d=2.75Å**		**Pb-benzene d=2.5Å**	
State	Energy (eV)	State	Energy (eV)	State	Energy (eV)	State	Energy (eV)	State	Energy (eV)	State	Energy (eV)
$\mathbf{^{3}P_{0}}$	0.00	$\mathbf{1A_1}$	0.00	$\mathbf{1A_1}$	0.00	$\mathbf{1A_1}$	0.00	$\mathbf{1A_1}$	0.00	$\mathbf{1A_1}$	0.00
		$\mathbf{1E_1}$	0.85	$\mathbf{1E_1}$	0.84	$\mathbf{1E_1}$	0.74	$\mathbf{1E_1}$	0.68	$\mathbf{1E_1}$	0.59
$\mathbf{^{3}P_{1}}$	0.86	$\mathbf{1E_1}$	0.85	$\mathbf{1E_1}$	0.84	$\mathbf{1E_1}$	0.74	$\mathbf{1E_1}$	0.68	$\mathbf{1E_1}$	0.59
		$\mathbf{1A_2}$	0.89	$\mathbf{1A_2}$	0.93	$\mathbf{1A_2}$	1.10	$\mathbf{1A_2}$	1.18	$\mathbf{1E_2}$	1.23
		$\mathbf{1E_2}$	1.33	$\mathbf{1E_2}$	1.33	$\mathbf{1E_2}$	1.31	$\mathbf{1E_2}$	1.28	$\mathbf{1E_2}$	1.23
		$\mathbf{1E_2}$	1.33	$\mathbf{1E_2}$	1.33	$\mathbf{1E_2}$	1.31	$\mathbf{1E_2}$	1.28	$\mathbf{1A_2}$	1.27
$\mathbf{^{3}P_{2}}$	1.33	$\mathbf{2E_1}$	1.34	$\mathbf{2E_1}$	1.36	$\mathbf{2A_1}$	1.42	$\mathbf{2A_1}$	1.45	$\mathbf{2A_1}$	1.47
		$\mathbf{2E_1}$	1.34	$\mathbf{2E_1}$	1.36	$\mathbf{2E_1}$	1.45	$\mathbf{2E_1}$	1.50	$\mathbf{2E_1}$	1.56
		$\mathbf{2A_1}$	1.34	$\mathbf{2A_1}$	1.36	$\mathbf{2E_1}$	1.45	$\mathbf{2E_1}$	1.50	$\mathbf{2E_1}$	1.56
		$\mathbf{2E_2}$	2.74	$\mathbf{2E_2}$	2.73	$\mathbf{2E_2}$	2.69	$\mathbf{2E_2}$	2.66	$\mathbf{2E_2}$	2.61
		$\mathbf{2E_2}$	2.74	$\mathbf{2E_2}$	2.73	$\mathbf{2E_2}$	2.69	$\mathbf{2E_2}$	2.66	$\mathbf{2E_2}$	2.61
$\mathbf{^{1}D_{2}}$	2.76	$\mathbf{3E_1}$	2.77	$\mathbf{3E_1}$	2.80	$\mathbf{3A_1}$	2.85	$\mathbf{3A_1}$	2.80	$\mathbf{3A_1}$	2.67
		$\mathbf{3E_1}$	2.77	$\mathbf{3E_1}$	2.80	$\mathbf{3E_1}$	2.86	$\mathbf{3E_1}$	2.88	$\mathbf{3E_1}$	2.87
		$\mathbf{3A_1}$	2.77	$\mathbf{3A_1}$	2.81	$\mathbf{3E_1}$	2.86	$\mathbf{3E_1}$	2.88	$\mathbf{3E_1}$	2.87
$\mathbf{^{1}S_{0}}$	4.10	$\mathbf{4A_1}$	4.11	$\mathbf{4A_1}$	4.14	$\mathbf{4A_1}$	4.25	$\mathbf{4A_1}$	4.32	$\mathbf{4A_1}$	4.41

Table S2. Excited-state energies and term symbols for isolated Pb and Pb-benzene complex at various distances calculated with the 4c-DHF method where c=25,000 a.u.

**Isolated Pb**		**Pb-benzene d=5Å**		**Pb-benzene d=4Å**		**Pb-benzene d=3Å**		**Pb-benzene d=2.75Å**		**Pb-benzene d=2.5Å**	
State	Energy (eV)	State	Energy (eV)	State	Energy (eV)	State	Energy (eV)	State	Energy (eV)	State	Energy (eV)
$\mathbf{^{3}P_{0}}$	0.00	$\mathbf{^{3}A_2}$	0.00	$\mathbf{^{3}A_2}$	0.00	$\mathbf{^{3}A_2}$	0.00	$\mathbf{^{3}A_2}$	0.00	$\mathbf{^{3}A_2}$	0.00
		$\mathbf{^{3}A_2}$	0.00	$\mathbf{^{3}A_2}$	0.00	$\mathbf{^{3}A_2}$	0.00	$\mathbf{^{3}A_2}$	0.00	$\mathbf{^{3}A_2}$	0.00
$\mathbf{^{3}P_{1}}$	0.00	$\mathbf{^{3}A_2}$	0.00	$\mathbf{^{3}A_2}$	0.00	$\mathbf{^{3}A_2}$	0.00	$\mathbf{^{3}A_2}$	0.00	$\mathbf{^{3}A_2}$	0.00
		$\mathbf{^{3}E_1}$	0.06	$\mathbf{^{3}E_1}$	0.17	$\mathbf{^{3}E_1}$	0.63	$\mathbf{^{1}E_2}$	0.87	$\mathbf{^{1}E_2}$	0.85
		$\mathbf{^{3}E_1}$	0.06	$\mathbf{^{3}E_1}$	0.17	$\mathbf{^{3}E_1}$	0.63	$\mathbf{^{1}E_2}$	0.87	$\mathbf{^{1}E_2}$	0.85
		$\mathbf{^{3}E_1}$	0.06	$\mathbf{^{3}E_1}$	0.17	$\mathbf{^{3}E_1}$	0.63	$\mathbf{^{3}E_1}$	0.88	$\mathbf{^{3}E_1}$	1.21
$\mathbf{^{3}P_{2}}$	0.00	$\mathbf{^{3}E_1}$	0.06	$\mathbf{^{3}E_1}$	0.17	$\mathbf{^{3}E_1}$	0.63	$\mathbf{^{3}E_1}$	0.88	$\mathbf{^{3}E_1}$	1.21
		$\mathbf{^{3}E_1}$	0.06	$\mathbf{^{3}E_1}$	0.17	$\mathbf{^{3}E_1}$	0.63	$\mathbf{^{3}E_1}$	0.88	$\mathbf{^{3}E_1}$	1.21
		$\mathbf{^{3}E_1}$	0.06	$\mathbf{^{3}E_1}$	0.17	$\mathbf{^{3}E_1}$	0.63	$\mathbf{^{3}E_1}$	0.88	$\mathbf{^{3}E_1}$	1.21
		$\mathbf{^{1}E_2}$	0.91	$\mathbf{^{1}E_2}$	0.91	$\mathbf{^{1}E_2}$	0.89	$\mathbf{^{3}E_1}$	0.88	$\mathbf{^{3}E_1}$	1.21
		$\mathbf{^{1}E_2}$	0.91	$\mathbf{^{1}E_2}$	0.91	$\mathbf{^{1}E_2}$	0.89	$\mathbf{^{3}E_1}$	0.88	$\mathbf{^{3}E_1}$	1.21
$\mathbf{^{1}D_{2}}$	0.98	$\mathbf{^{1}E_1}$	0.97	$\mathbf{^{1}E_1}$	1.08	$\mathbf{^{1}A_1}$	1.45	$\mathbf{^{1}A_1}$	1.52	$\mathbf{^{1}A_1}$	1.56
		$\mathbf{^{1}E_1}$	0.97	$\mathbf{^{1}E_1}$	1.08	$\mathbf{^{1}E_1}$	1.47	$\mathbf{^{1}E_1}$	1.68	$\mathbf{^{1}E_1}$	1.95
		$\mathbf{^{1}A_1}$	0.99	$\mathbf{^{1}A_1}$	1.11	$\mathbf{^{1}E_1}$	1.47	$\mathbf{^{1}E_1}$	1.68	$\mathbf{^{1}E_1}$	1.95
$\mathbf{^{1}S_{0}}$	2.27	$\mathbf{^{1}A_1}$	2.32	$\mathbf{^{1}A_1}$	2.41	$\mathbf{^{1}A_1}$	2.86	$\mathbf{^{1}A_1}$	3.19	$\mathbf{^{1}A_1}$	3.66

Table S3. Excited-state energies and term symbols for isolated Pb and Pb-benzene complex at various distances calculated with the ZORA-CIS method

**Isolated Pb**		**Pb-benzene d=5Å**		**Pb-benzene d=4Å**		**Pb-benzene d=3Å**		**Pb-benzene d=2.75Å**		**Pb-benzene d=2.5Å**	
State	Energy (eV)	State	Energy (eV)	State	Energy (eV)	State	Energy (eV)	State	Energy (eV)	State	Energy (eV)
$\mathbf{^3P_0}$	0.00	$\mathbf{1A_1}$	0.00	$\mathbf{1A_1}$	0.00	$\mathbf{1A_1}$	0.00	$\mathbf{1A_1}$	0.00	$\mathbf{1A_1}$	0.00
	0.52	$\mathbf{1E_1}$	0.51	$\mathbf{1E_1}$	0.50	$\mathbf{1E_1}$	0.43	$\mathbf{1E_1}$	0.39	$\mathbf{1E_1}$	0.32
$\mathbf{^3P_1}$		$\mathbf{1E_1}$	0.51	$\mathbf{1E_1}$	0.50	$\mathbf{1E_1}$	0.43	$\mathbf{1E_1}$	0.39	$\mathbf{1E_1}$	0.32
		$\mathbf{1A_2}$	0.54	$\mathbf{1A_2}$	0.58	$\mathbf{1A_2}$	0.76	$\mathbf{1A_2}$	0.85	$\mathbf{1E_2}$	0.93
	1.09	$\mathbf{1E_2}$	1.08	$\mathbf{1E_2}$	1.07	$\mathbf{1E_2}$	1.02	$\mathbf{1E_2}$	0.99	$\mathbf{1E_2}$	0.93
		$\mathbf{1E_2}$	1.08	$\mathbf{1E_2}$	1.07	$\mathbf{1E_2}$	1.02	$\mathbf{1E_2}$	0.99	$\mathbf{1A_2}$	0.95
$\mathbf{^3P_2}$		$\mathbf{2E_1}$	1.09	$\mathbf{2E_1}$	1.11	$\mathbf{2E_1}$	1.20	$\mathbf{2A_1}$	1.24	$\mathbf{2A_1}$	1.26
		$\mathbf{2E_1}$	1.09	$\mathbf{2E_1}$	1.11	$\mathbf{2E_1}$	1.20	$\mathbf{2E_1}$	1.26	$\mathbf{2E_1}$	1.32
		$\mathbf{2A_1}$	1.09	$\mathbf{2A_1}$	1.11	$\mathbf{2A_1}$	1.20	$\mathbf{2E_1}$	1.26	$\mathbf{2E_1}$	1.32

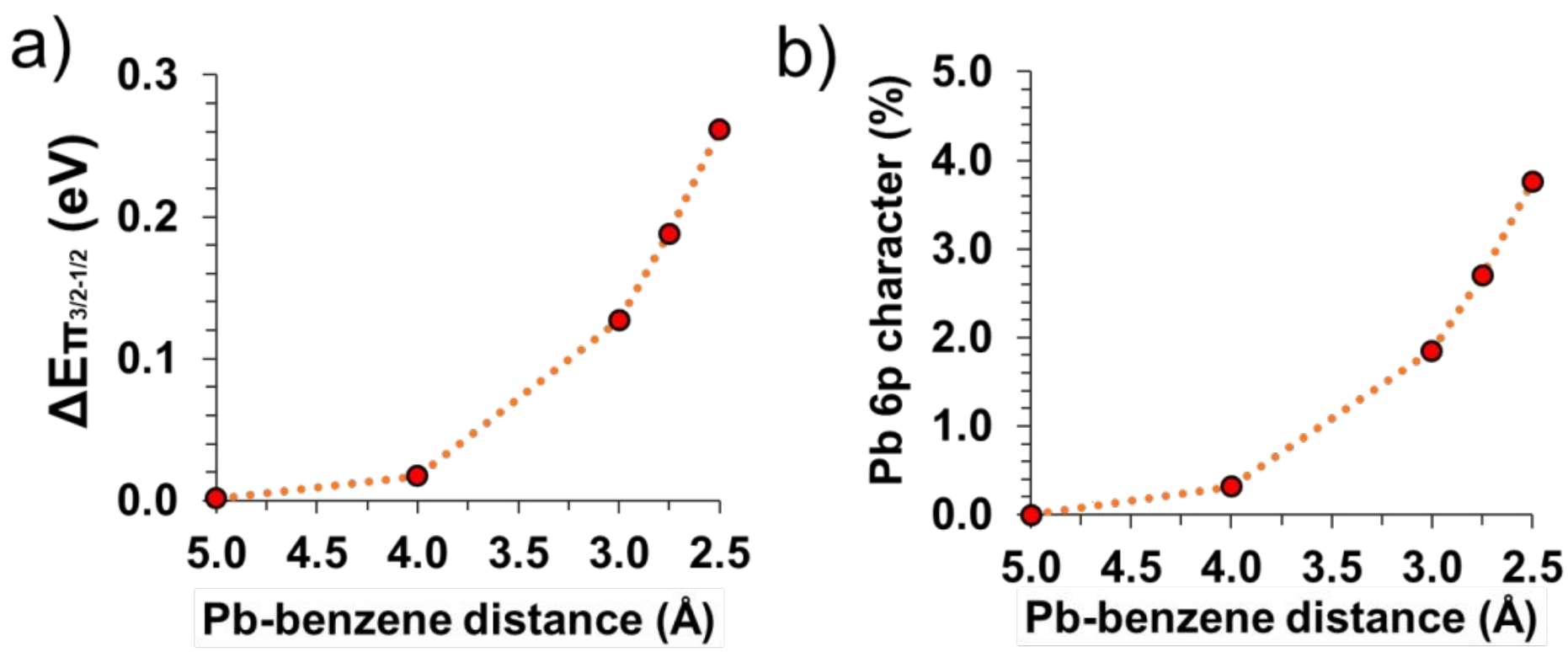

Figure S1. a) The SO splitting of π levels ($1e_{1/2}$-$1e_{3/2}$) originating dominantly from benzene upon mixing with Pb 6p orbitals. b) The percentage of Pb 6p character for the resulting $1e_{1/2}$-$1e_{3/2}$ MOs.

Table S4. Orbital energies and SO splittings for the benzene-originating π levels of Pb-benzene complex.

		**ZORA/CIS (j-j limit)**	**DHF-4c (j-j limit)**	**DHF-4c (configuration-mixed)**
Distance	Label	Energy (eV)	Energy (eV)	Energy (eV)
**5 Å**	$1e_{1/2}$	-9.28	-9.22	-9.22
	$1e_{3/2}$	-9.28	-9.22	-9.22
	Δ_{so}	0.00	0.00	0.00
**4 Å**	$1e_{1/2}$	-9.38	-9.32	-9.34
	$1e_{3/2}$	-9.36	-9.30	-9.34
	Δ_{so}	0.02	0.02	0.00
**3 Å**	$1e_{1/2}$	-9.90	-9.82	-9.87
	$1e_{3/2}$	-9.77	-9.70	-9.86
	Δ_{so}	0.13	0.13	0.01
**2.75 Å**	$1e_{1/2}$	-10.19	-10.11	-10.18
	$1e_{3/2}$	-10.00	-9.92	-10.15
	Δ_{so}	0.19	0.19	0.02
**2.5 Å**	$1e_{1/2}$	-10.59	-10.50	-10.62
	$1e_{3/2}$	-10.33	-10.24	-10.57
	Δ_{so}	0.26	0.26	0.05

Basis set for DHF calculations

Pb basis

s functions	p functions	d functions	f functions
**Block 1 (20 primitives)**	**Block 1 (20 primitives)**	**Block 1 (17 primitives)**	**Block 1 (11 primitives)**
6.03798479E+07	4.48876065E+07	3.29356064E+04	1.00520285E+03
1.60690745E+07	1.16821975E+07	8.06417412E+03	3.41495533E+02
5.50158015E+06	3.41127699E+06	2.74121675E+03	1.45275798E+02
2.09541152E+06	1.08859283E+06	1.11092616E+03	6.89365180E+01
8.73747986E+05	3.72414988E+05	5.02922099E+02	3.45975469E+01
3.85240024E+05	1.35125079E+05	2.44704323E+02	1.78845449E+01
1.78043163E+05	5.17028736E+04	1.25285235E+02	9.22196908E+00
8.50480279E+04	2.08204042E+04	6.61554572E+01	4.64266605E+00
4.17656436E+04	8.82465553E+03	3.57496541E+01	2.16866137E+00
2.09673002E+04	3.93487429E+03	1.93406179E+01	8.02167191E-01
1.07363806E+04	1.84103284E+03	1.02760058E+01	5.60408710E-01
5.59612309E+03	8.99380189E+02	5.42011456E+00	
2.96529042E+03	4.55452747E+02	2.76252810E+00	
1.59507762E+03	2.37621095E+02	1.35839970E+00	
8.69510257E+02	1.26467890E+02	6.38064514E-01	
4.79292554E+02	6.77241843E+01	2.81842020E-01	
2.67978643E+02	3.71724457E+01	1.06118956E-01	
1.51962899E+02	2.03301293E+01		
8.69502226E+01	1.08287874E+01		
4.92454742E+01	5.78253851E+00		
**Block 2 (10 primitives)**	**Block 2 (6 primitives)**		
2.89250027E+01	2.93572621E+00		
1.68005206E+01	1.47773217E+00		
9.23723643E+00	7.01333423E-01		
5.22327683E+00	2.63794096E-01		
2.77804722E+00	1.04471072E-01		
1.49086619E+00	3.97592513E-02		
7.65783736E-01			
2.97829937E-01			
1.36025214E-01			
5.89616920E-02			

C basis

s functions	p functions	d functions
8806.653	32.6921	0.30
1319.282	7.4726	0.10
299.519	2.2392	
84.3388	0.7728	
27.1337	0.2748	
9.4934	0.0958	
3.4863		
1.0175		
0.3937		
0.1343		

H basis

s functions	p functions
33.64000	1.100000
5.058000	
1.147000	
0.321100	
0.101300	
